# MRSA Carriage in Community Outpatients: A Cross-Sectional Prevalence Study in a High-Density Livestock Farming Area along the Dutch-German Border

**DOI:** 10.1371/journal.pone.0139589

**Published:** 2015-11-30

**Authors:** John Paget, Helen Aangenend, Malte Kühn, Jeannine Hautvast, Desiree van Oorschot, Alphons Olde Loohuis, Koos van der Velden, Alexander W. Friedrich, Andreas Voss, Robin Köck

**Affiliations:** 1 Radboud University Medical Center, Department of Primary and Community Care, Nijmegen, the Netherlands; 2 Netherlands Institute for Health Services Research (NIVEL), Utrecht, the Netherlands; 3 GGD Gelderland-Zuid (Municipal Health Service Gelderland-South), Nijmegen, the Netherlands; 4 University Hospital Münster, Institute of Hygiene, Münster, Germany; 5 University of Groningen, University Medical Center Groningen, Department of Medical Microbiology, Groningen, the Netherlands; 6 Canisius-Wilhelmina Hospital, Department of Clinical Microbiology and Infectious Diseases, Nijmegen, the Netherlands; 7 Radboud University Medical Center, Department of Medical Microbiology, Nijmegen, the Netherlands; Rockefeller University, UNITED STATES

## Abstract

**Objectives:**

MRSA poses a considerable public health threat to the community. The objectives of this study were to assess the prevalence of MRSA carriage and determine factors that were associated with MRSA carriage among outpatients who had used antibiotics in the previous three months and who lived in a high-density livestock farming area along the Dutch-German border.

**Methods:**

Cross-sectional prevalence study carried out between November 2011 and June 2012. Nasal swabs and questionnaires were collected in patients (>4 years) who had used antibiotics in the previous three months from twelve Dutch General Practitioners (GPs), seven German GPs and two German outpatient urologists. To assess nasal carriage, swabs were analyzed using selective MRSA agars after broth enrichment. MRSA positive samples were *spa* typed.

**Results:**

Data were collected from 513 GP outpatients in the Netherlands, 261 GP outpatients in Germany and 200 urologist outpatients in Germany. The overall prevalence of MRSA carriage was 0.8%, 1.1% and 2.0%, respectively. In the GP outpatient populations, the prevalence was similar in both countries (0.8% and 1.1%, respectively, p = 0.879), all *spa* types were indicative for livestock-associated MRSA (4xt011 in the Netherlands; 2xt034 and t011 in Germany) and being a farmer, living on or near (<5km) to a farm were associated with MRSA carriage. In the urologist outpatient population, the prevalence was higher (2.0%), all *spa* types were indicative for healthcare-associated MRSA (t068, t032, t003, t10231) and being a farmer, living on or near to a farm were factors not associated with MRSA carriage.

**Conclusions:**

The prevalence of MRSA carriage in these community outpatient populations along the Dutch-German border was low. There were striking similarities in livestock-associated MRSA carriage and clonal spread in the outpatient populations seeing their GP in both countries. In contrast, urologist outpatients in Germany were colonized with *spa* types indicative of healthcare-associated MRSA.

## Introduction

The epidemiology of Methicillin-resistant *Staphylococcus aureus* (MRSA) in Europe has changed since the 1990s. Next to healthcare-associated (HA)- or community-acquired (CA) strains, livestock-associated (LA) MRSA have emerged as a third entity. The most important LA-MRSA circulating among humans belong to clonal complex 398 (CC398) as defined by multilocus sequence typing (MLST), with the reservoir for this pathogen mainly found in pigs and cattle [1], and more recently, in poultry [2]. Using *S*. *aureus* protein A (*spa*) sequence typing, MRSA associated with CC398 are characterized by *spa* types t011, t034, t108 and close relatives [3]. Persons with LA-MRSA were first detected in the Netherlands in 2003 [4] and soon after in Germany [5].

Several studies have shown that persons in direct contact with livestock, especially pigs and veal calves, have an increased risk for MRSA [3,4,6]. General practitioners (GPs) are concerned that some common infections (e.g. skin and soft-tissue infections and urinary tract infections) could increasingly be caused by MRSA and thus become more difficult to treat [7] as described after the emergence of CA-MRSA in the USA [8]. As MRSA is associated with higher mortality and costs, it is of utmost importance that the spread of MRSA is controlled and prevented [9,10].

**Fig 1:**
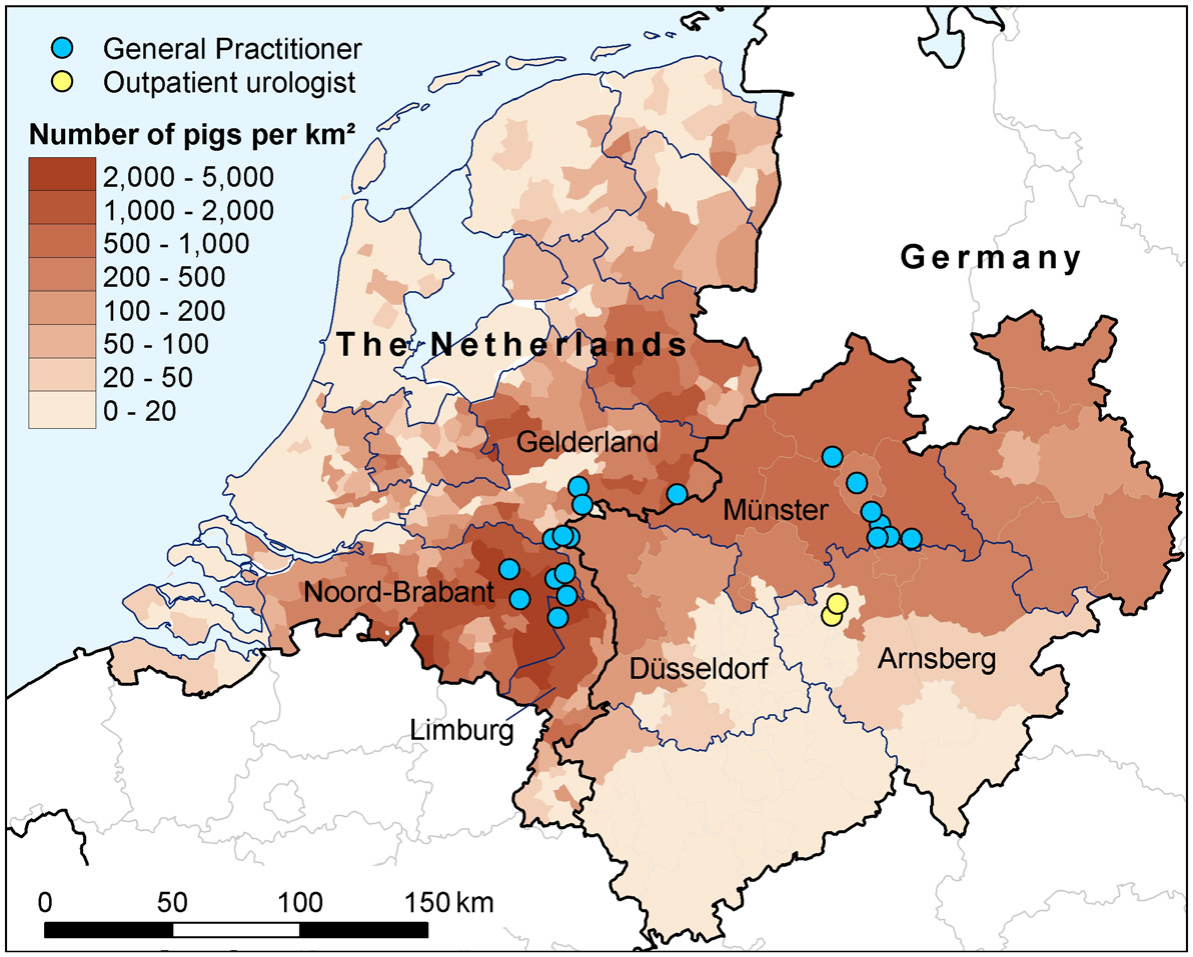
Map of the Netherlands and the German federal state of North Rhine-Westphalia: pig-density rates (pigs per km^2^) and location of GPs and outpatient urologists. Note: The pig-density data for the Netherlands is from 2012 and for North Rhine-Westphalia it is from 2010 (the most recent data available for both countries).

The objective of this study was to assess the prevalence and factors that were associated with MRSA carriage among persons who visited their GP and had used antibiotics in the previous three months. To our knowledge this is the first study carried out in persons who had used antibiotics, and we expected a higher prevalence of MRSA as studies have shown a causal link between bacterial resistance and antibiotic usage at an individual patient level [7, 11], with the greatest effect in the month immediately after treatment and probable persistence for up to 12 months [7]. The research was carried out in an area of high density livestock farming (see Fig 1), which increased the risk of MRSA carriage [5,6], specifically, along the border of the Netherlands (Gelderland, Brabant, Limburg; Dutch region) and Germany (Münsterland, Arnsberg in North Rhine-Westphalia; German region).

## Methods

A cross-sectional prevalence study was carried out in each region between November 2011 and June 2012.

### Ethics Statement

In the Netherlands, the study protocol was submitted to the Medical Ethical Review Board of the Radboud University Nijmegen Medical Center, which indicated that ethical review was not required (registration number 2011/129) and in Germany it was approved by the Medical Ethical Commission of the Ärztekammer Westfalen-Lippe (2006-268-f-S). All participants provided written informed consent to participate in the study. For children participating in the study, written informed consent was obtained from a parent or guardian. The ethics committees approved this consent procedure.

### Sample size calculation

Our objectives was to determine a 50% difference in the prevalence of MRSA carriage rates between the two regions. When planning the study, we did not know the prevalence of MRSA and we assumed that persons who had used antibiotics in the previous 3 months would have a higher rate of carriage [7,11]. To determine a 50% difference in the prevalence of MRSA carriage rates between the two regions (assuming a 4% carriage rate in the Dutch region) with a 95% precision, 1,000 patients were required in each region.

### Recruitment of the participating GPs and outpatient urologists

We assumed it would be feasible to collect 100 nasal swabs per GP and therefore included 10 GPs in each region. In the Netherlands, the GPs were recruited via the GP network of the Department of Primary and Community Care at the Radboud university medical center (3 GPs) and by randomly contacting GPs by telephone in the three provinces along the Dutch-German border (9 GPs).

In Germany, the physicians in primary care were asked to participate via the Regional Organization for Physicians in Primary Care; all physicians who responded (7 GPs and 2 urologists) were contacted by the study centre based at the University Hospital Münster and were included without further selection. The two outpatient urologists were included in the study as it was difficult to recruit GPs in the German study region and, in contrast to the Netherlands, access to specialized outpatient urologist care in Germany is available to all patients with urological symptoms (a referral by the patient’s GP is not required).

### Data collection

The persons included in the study were four years and older, had used antibiotics during the previous three months, were not terminally ill and were not immuno-compromised. The GPs / outpatient urologists used the patient’s medical dossier to assess whether patients had received an antibiotic in the previous three months and these persons were invited to participate in the study. A person who was prescribed antibiotics at the consultation, but who had not used antibiotics during the previous three months, was excluded from the study.

All persons who participated in the study were recruited by the practicing GP/outpatient urologist and met the study inclusion criteria. Once the GP/outpatient urologist had assessed whether the patient was willing to enter the study, the person was asked to complete an informed consent form. The GP, outpatient urologist, nurse or practice researcher then took a swab from the anterior nostrils and completed a short questionnaire (8 questions) together with the participant (S1 File). The first question covered the type of antibiotics used in the previous three months and the medical indication for prescribing them; the other questions covered basic demographic information and risk factors for MRSA carriage. No data was collected on patients who refused to participate in the study but GPs/outpatient urologists were asked to continue the study until they reached a target number of 100 patients. All patient-related data on the swabs and questionnaire were anonymized using a study code only known to the GP/outpatient urologist.

### Collection of the nose swabs and study questionnaires

Nasal swabs (a dry swab applied to both nostrils) and questionnaires were collected in persons treated by the twelve Dutch GPs, seven German GPs and two German outpatient urologists. The GPs participating in the study in both regions had difficulties reaching the target of 100 nose swabs during the data collection period as fewer patients than planned were eligible for inclusion in the study and organizational issues (e.g. the active inclusion of patients by GPs tailored off over time). The study finally collected 513 nose swabs in the Netherlands (range: 7 to 79 per GP) and 461 in Germany (range: 8 to 70 per GP and 100 for the two outpatient urologists).

### Microbiological testing

The swabs and questionnaires were sent to a central laboratory in each country for microbiological testing and were then returned to the study co-ordination centre at the Radboud university medical center. The microbiological laboratories in the Netherlands and Germany used a harmonized diagnostic protocol: swabs were enriched overnight at 36°C in Mueller-Hinton broth containing 6.5% sodium chloride; 10 μl of this sample were streaked onto a chromogenic medium for the detection of MRSA (bioMérieux, Marcy l’Etoile, France) as well as Columbia blood agar (for the detection of methicillin-susceptible *S*. *aureus* (MSSA)) and incubated for 18-24h (36°C). Suspicious colonies were confirmed as MRSA using VITEK-2 automated systems (with EUCAST breakpoints for susceptibility testing), *nuc*-PCR and *mecA*-PCR. All MRSA isolates were subjected to *S*. *aureus* protein A (*spa*) sequence-typing [12]. In Germany, all swabs were tested for MRSA and MSSA, whilst in the Netherlands all swabs were tested for MRSA (513 swabs) and testing for MSSA was only added to the routine testing protocol at the end of the study (82 swabs).

### Statistical analysis

The statistical analysis was carried out by IBM SPSS statistics 2.0 with descriptive and bivariate statistics. P-values were derived from Chi-square tests.

## Results

A total of 974 persons were included in the study and 11 (1.1%) were MRSA carriers. The prevalence of MRSA carriage was 0.8% in the Dutch GP outpatients, 1.1% in the German GP outpatients and 2.0% in the German urologist outpatients (none of these differences were significant: p = 0.879, p = 0.714, p = 0.319). The prevalence of *S*. *aureus* carriage (MSSA and MRSA) was 31% in the Netherlands (based on the sub-sample of 82 swabs) and 24% in Germany (based on all 461 swabs).

In the Dutch region the median age of all study participants was higher than in the German region (58.3 versus 53.6 years) and this difference was especially strong for the GP outpatients (58.3 versus 50.3). More females than men participated in the study in both countries and more than 10% of persons were healthcare workers among the GP outpatients (Table 1). Among GP outpatients, the percentage of farmers (3.3% and 3.8%, respectively) and patients who were hospitalized in the previous six months was very similar. As expected, the German urologist outpatients mainly saw patients who had received antibiotics to treat a urinary tract infection (98.5%) in the previous three months. None of the urologist outpatients were farmers.

**Table 1:** Characteristics of participating patients: Dutch region (Gelderland, Brabant, Limburg) and German region (North Rhine-Westphalia).

Characteristic	Description	Dutch region	German region		Total
		GP outpatients	GP outpatients	Urologist outpatients	
**Total**	**Number**	**513**	**261**	**200**	**461**
Mean age	Years	58.3	50.3	57.8	53.6
Female	Yes	64.9%	66.3%	60.0%	63.6%
Healthcare worker	Yes	11.9%	15.7%	5.5%	11.3%
Farmer	Yes	3.3%	3.8%	0.0%	2.2%
Hospitalized last 6 months	Yes	15.8%	16.1%	30.0%	22.1%
MRSA positive	Yes	0.8%	1.1%	2.0%	1.5%
Health reason for which antibiotics were prescribed in previous three months:	Urinary Tract Infection	23.6%	16.5%	98.5%	
	Respiratory Infection	23.6%	16.5%	98.5%	52.1%
	Sinusitis/pharyngitis/laryngitis	35.6%	44.8%	0.5%	25.6%
	Others	10.4%	26.4%	0.0%	15.0%
		30.3%	12.3%	1.0%	7.4%
Antibiotic^1^ prescribed in previous three months:	Broad-spectrum penicillin	11.1%	5.7%	0.5%	
	Doxycycline/tetracycline	29.3%	6.1%	2.5%	3.5%
	TMP, SMZ and TMP/SMZ	4.6%	5.7%	8.5%	4.6%
	Macrolides	7.9%	27.6%	0.0%	6.9%
	Small-spectrum penicillin	24.6%	27.6%	1.0%	15.6%
	Fluoroquinolones	21.4%	12.6%	75.0%	16.1%
	Cephalosporines	0.2%	10.0%	10.5%	
	Others	0.6%	4.6%	2.0%	3.5%

^1^ Based on the first-reported antibiotic (93% of patients reported only one antibiotic in the previous 3 months): Broad-spectrum penicillin (amoxicillin/clavulanic acid); Small-spectrum penicillin (benzylpenicillin, amoxicillin, feneticillin, flucloxacillin); Cephalosporines (cefuroxime, cefpodoxime, ceftibuten, cefixime), TMP (trimethoprim); SMZ (sulfamethoxazole)

Table 2 shows the prevalence of MRSA carriage stratified by country and factors associated with MRSA carriage. Only factors that were significant are presented in the Table and we present some grouped categories due to the small number of MRSA cases. Among the GP outpatients, 1) being a Farmer; 2) being a Farmer and/or Living on a farm; 3) being a Farmer and/or Living on a farm and/or Living near to a farm (<5 km), were factors that were significantly associated with MRSA carriage in both countries. We could not identify any factor in the questionnaire that was significantly associated with MRSA carriage in the urologist outpatients in Germany, but this may have been a result of the smaller sample size in this patient group.

**Table 2:** Prevalence of MRSA carriage by population group and factors associated with MRSA carriage^1^: Dutch region (Gelderland, Brabant, Limburg) and German region (North Rhine-Westphalia).

Factor		Dutch region—GP^2^ outpatients			German region—GP outpatients			German region—urologist outpatients			Germany total
		N	MRSA positive n (%)	p-value	N	MRSA positive n (%)	p-value	N	MRSA positive n (%)	p-value	MRSA positive n (%)
**Total**		**513**	**4 (0.8)**		**261**	**3 (1.1)**		**200**	**4 (2.0)**		**7 (1.5)**
Farmer^3^	Yes	17	3 (17.6)	**<0.000**	10	2 (20.0)	**0.002**	0	0 (0.0)	-	2 (20.0)
	No	486	1 (0.2)		251	1 (0.4)		200	4 (2.0)		5 (1.1)
Farmer / Lives on a farm^3^	Yes	26	3 (11.5)	**<0.000**	20	2 (10,0)	**0.009**	1	0	0.840	2 (9.5)
	No	451	1 (0.2)		241	1 (0.4)		199	4 (2.0)		5 (1.1)
Farmer / Lives on a farm / Lives near to a farm (<5km)^3^	Yes	220	4 (1.8)	**0.011**	71	3 (4.2)	**0.005**	13	1 (7.7)	0.234	4 (4.8)
	No	267	0 (0.0)		190	0 (0.0)		187	3 (1.6)		3 (0.8%)

^1^ Data are only presented for significant factors collected in the study questionnaire (S1 File)

^2^ GPs: General Practitioners

^3^ Due to missing responses, the numbers do not always add up to the total

We also assessed whether MRSA carriage was associated with an antibiotic type or antibiotic usage in the previous three months (calculated by multiplying the dose by the treatment duration and the number of tablets per day; with usage classified as ‘high’ or ‘low’ based on a 2500 mg cut-off). We found no significant associations for the antibiotic types. For antibiotic usage, we found a significant association with MRSA carriage for GP patients in the Netherlands (p = 0.033), but not for GP (p = 0.169) or urologist (p = 0.140) patients in Germany.

Table 3 presents the characteristics of all MRSA cases detected in this study. In the Dutch region we found only *spa* types indicative for LA-MRSA CC398 (n = 4; t011), in the German region four HA- (t003, t032, t068 and t10231) and three LA-MRSA (t011, n = 1; t034, n = 2). All cases among GP outpatients were associated with LA-MRSA and all cases in the urologist outpatients in Germany were caused by HA-MRSA strains, known to circulate in the German healthcare system [13]. In total, of seven persons with LA-MRSA, five were farmers and all were GP outpatients. Three of four German urologist outpatients colonized with HA-MRSA strains were either healthcare workers or had been hospitalized within the past six months before the investigation.

**Table 3:** MRSA cases by spa-type and region: Dutch region (Gelderland, Brabant and Limburg) and German region (North Rhine-Westphalia).

Country region and type of physicians	MRSA spa type	Sex/age	Farmer/ Health care worker	Contact with living animals	Lives on/near farm	Hospitalization in the last six months	Health reason for which antibiotics were prescribed (previous three months)	Antibiotic group^2^	Antibiotic specification
**Dutch region: GP** ^1^ **outpatients (n = 4)**	t011	M/80	No	No	Yes	Yes	Respiratory tract infection	Broad-spectrum penicillin	Amoxicillin/Clavulanic acid
	t011	F/40	Farmer	No	Yes	No	Skin and soft tissue infection	Broad-spectrum penicillin	Amoxicillin/Clavulanic acid
	t011	F/69	Farmer	Cattle	Yes	No	Respiratory tract infection	Small-spectrum penicillin	Amoxicillin
	t011	F/69	Farmer	Cattle	Yes	No	Respiratory tract infection	Broad-spectrum penicillin	Amoxicillin/Clavulanic acid
**German Region: GP outpatients (n = 3)**	t034	F/29	HCW	No	Yes	No	Sinusitis/Pharyngitis/Laryngitis	Small-spectrum penicillin	Amoxicillin
	t034	M/40	Farmer/HCW	Pigs	Yes	No	Sinusitis/Pharyngitis/Laryngitis	Small-spectrum penicillin	Amoxicillin
	t011	F/75	Farmer	Pigs	Yes	No	Respiratory tract infection	Small-spectrum penicillin	Amoxicillin
**German Region: Urologist outpatients (n = 4)**	t032	F/73	No	No	No	Yes	Urinary tract infection	Fluoroquinolones	Nitrofurantoin
	t10231	F/43	HCW	No	No	Yes	Urinary tract infection	Cepahlosporin	Ceftibuten
	t003	M/69	No	No	No	Yes	Urinary tract infection	Cepahlosporin	Cefuroxime
	t068	F/66	No	No	Yes	No	Urinary tract infection	TMP/SMZ	Cotrimoxazole

^1^ GP: General Practitioner

^2^ Based on the first-reported antibiotic (93% of patients reported only one antibiotic in the previous 3 months): Broad-spectrum penicillin (amoxicillin/clavulanic acid); Small-spectrum penicillin (benzylpenicillin, amoxicillin, feneticillin, flucloxacillin); Cephalosporines (cefuroxime, cefpodoxime, ceftibuten, cefixime); TMP (trimethoprim) / SMZ (sulfamethoxazole)

In the Netherlands the four cases of MRSA were found among three GPs, with one GP having two cases of MRSA (2 out of a total of 55 nasal swabs). In Germany, three cases of MRSA came from one GP (who reported a total of 63 nasal swabs), 3 cases came from one urologist (100 nasal swabs) and one case from the other urologist (100 nasal swabs). We were not able to link the MRSA cases epidemiologically (e.g. family members) as the data were anonymous.

## Discussion

To our knowledge, this is the first study that has focused on the prevalence of MRSA carriage in community outpatients who have used antibiotics in the last three months. Despite sampling from this population and persons who lived in high-density livestock areas, the prevalence of MRSA carriage was low in both countries: 0.8% in the Dutch GP patients, 1.1% in the German GP outpatients and 2.0% in German urologist outpatients. The prevalence of *S*. *aureus* carriage (MSSA and MRSA) was 31% in the Netherlands and 24% in Germany, which is comparable to the average prevalence in the general population and confirms appropriate technical retrieval of the nasal swabs [10, 14].

We expected a higher prevalence of MRSA carriage in the German region, as there is a higher density of antibiotic use in outpatient care in Germany (14.9 Daily Defined Doses per 1,000 inhabitants per day (DID) compared to 11.4 DID in the Netherlands [15]), there is a more frequent use of fluoroquinolones among German patients which might be associated with increased selective pressure [16], there are more aggressive MRSA search and destroy policies implemented in Dutch healthcare facilities [10], and there is a higher density of inpatient care in Germany which facilitates the inter-facility spread of MRSA (8.2 patient beds per 1000 inhabitants in Germany vs. 4.7/1000 inhabitants in the Netherlands) [17]. This has been supported by studies which have estimated that the incidence of episodes of MRSA-bacteremia was 57.6 per 1,000,000 inhabitants in Germany (North Rhine-Westhalia) compared to 1.8 episodes per 1,000,000 inhabitants in the Netherlands [18]. Moreover, the proportion of MRSA on all *S*. *aureus* isolated from blood cultures was 15.4% in Germany and 1.3% in the Netherlands in 2012 [19]. Surprisingly, there were no significant differences in MRSA carriage between the outpatient groups in the two countries, and when we only looked at GP patients the MRSA rates were very similar: 1.1% in Germany compared to 0.8% in the Netherlands (p = 0.879).

Our findings show similarities and differences with other MRSA-prevalence studies that have been carried out in the Netherlands and Germany in different study populations. The prevalence of MRSA carriage detected in our study was comparable to the prevalence among patients at admission to hospitals in a Dutch-German study in 2006 (0.5/100 patients in the Netherlands and 1.6/100 patients in Germany) [20], but was higher compared to data from a recent Dutch study which showed an MRSA prevalence of 0.11% at hospital admission [21]. MRSA carriage was also higher than a study undertaken in 2010 among healthy persons attending their GPs in 9 countries across Europe, but this was expected as our study was carried out among persons who had used antibiotics in the previous three months. Among healthy persons attending their GP the prevalence rate was 0.2% in the Netherlands (n = 1073) and ranged from 0% in Sweden to 0.4% in Belgium, Croatia, France and the United Kingdom (no data for Germany) [14].

The factors that were significantly associated with MRSA colonization in both countries in the GP outpatient populations were: being a farmer and living on or near (<5km) to a farm. For example, persons who were farmers and/or lived on a farm had the highest MRSA prevalence on both sides of the border: 11.5% in the Dutch region (3 out of 26) and 10.0% in the German region (2 out of 20) (Table 2). These results are similar to Van Cleef in the Netherlands (26.5% of persons who reported contact with livestock were positive for MRSA) [6], and have been shown in various studies before [22,23].

A number of review studies have found associations between antimicrobial consumption and MRSA carriage that are dose-dependent [24, 25]. Our study found an association between antibiotic usage (‘high’ usage (>2,500 mg) in the previous three months) and MRSA carriage for GP patients in the Netherlands but not for the patients in Germany, although this might have been linked to the small sample sizes in the two German patient groups (n = 261 for the GP patients and 200 for the urologist patients).

Our study also confirmed that persons carrying MRSA *spa* types indicative for the LA-MRSA clonal lineage CC398 (t011, t034) mostly (4 out of 7), but not exclusively, had a history of livestock contact. To some extent this confirmed recent findings that there is a certain proportion of patients colonized with LA-MRSA for whom acquisition via direct animal contact seems unlikely [5, 23]. For these patients, other sources like nosocomial acquisition (in this study one healthcare worker carried MRSA t034) or indirect transmission routes should be considered.

The higher rates of MRSA carriage in the German urologist outpatients, the different factors associated with MRSA carriage, and the different clonal spread in this population (all four strains were indicative of HA-MRSA), might be explained by a number of factors. The urologist outpatients come from a different area of North-West Germany, with lower levels livestock farming (see Fig 1). The urologist outpatients are also a different population compared to the GP outpatients in Germany: there were no farmers amongst these patients (versus 3.8% in GP outpatients) and, importantly, 30% had been hospitalised in the previous 6 months (versus 16.1% for the GP outpatients; Table 1).

Our study had a number of limitations. One important limitation was that the study was powered for 1000 patients in each country and we were only able to recruit 513 patients in the Netherlands and 461 patients in Germany. This limited our ability to show a significant difference in the prevalence of MRSA in the two countries and, due to the very small number of MRSA patients, prevented a more detailed statistical analysis (using multivariate analytical approaches) to assess the factors associated with MRSA carriage. We also found that the outpatient population participating in this study, i.e. outpatients with previous antibiotic use, was not representative for the community. For example, the age of participants (58 years on the Dutch, 50 and 58 years on the German side), was older than expected for the mean age of persons in the community and 11.9% of the Dutch and 15.7% of the German (GP outpatients) participants worked in the healthcare sector which exceeds national statistical data (Netherlands 3.5% [26] and Germany 6% [27]). Therefore, the comparability to other MRSA prevalence studies performed in the community is limited. Finally, our results for outpatient urologists in Germany are based on patients from just two practices in an area of North Rhine-Westphalia with lower pig-density rates. This limits the generalizability of the findings and comparability of the data to the GP patients.

## Conclusions

In conclusion, despite sampling from a population that used antibiotics in the previous three months and lived in high-density livestock farming areas, the prevalence of MRSA carriage was low in the border regions of both countries. This is a positive public health finding, supplemented by the fact that it appears that we did not find an increase in the MRSA carriage in the Netherlands since an earlier study carried out in 2008–09 (2.4%) [6]. An important finding in the outpatient GP populations was that all *spa* types were indicative of livestock-associated MRSA and that the factors associated with carriage were being a farmer, and living on or near to a farm. In contrast, all MRSA *spa* types were indicative of healthcare-associated MRSA in the urologist outpatient population in Germany. Considering these results are only based on data from two urologists, their generalizability needs to be treated with care, but we recommend further research regarding the spread and risk factors for MRSA in this specific outpatient population.

## Supporting Information

S1 File Study questionnaire. (DOC)

## References

[1] Graveland H, Wagenaar JA, Heesterbeek H, Mevius D, van Duijkeren E, Heederik D. Methicillin Resistant Staphylococcus aureus ST398 in Veal Calf Farming: Human MRSA Carriage Related with Animal Antimicrobial Usage and Farm Hygiene. PLoS ONE 5(6): e10990 10.1371/journal.pone.0010990 20544020 PMC2882326

[2] Haenen AP, Geenen PL, Vesseur PC, Poldervaart ES, Bosch T, Huijsdens XW, et al Prevalence of livestock-associated MRSA in broiler flocks and risk factors for slaughterhouse personnel in The Netherlands. Epidemiol Infect. 2010 5;138(5):743–55. 10.1017/S0950268810000075 Epub 2010 Jan 29. 20109255

[3] van Cleef BA, Monnet DL, Voss A, Krziwanek K, Allerberger F, Struelens M, et al Livestock-associated methicillin-resistant *Staphylococcus aureus* in humans, Europe. Emerg Infect Dis. 2011 3;17(3):502–5. 10.3201/eid1703.101036 21392444 PMC3166010

[4] Voss A, Loeffen F, Bakker J, Klaassen C, Wulf M. Methicillin-resistant *Staphylococcus aureus* in pig farming. Emerg Infect Dis. 2005 12;11(12):1965–6 16485492 10.3201/eid1112.050428 PMC3367632

[5] Köck R, Harlizius J, Bressan N, Laerberg R, Wieler LH, Witte W, et al Prevalence and molecular characteristics of methicillin-resistant Staphylococcus aureus (MRSA) among pigs on German farms and import of livestock-related MRSA into hospitals. Eur J Clin Microbiol Infect Dis. 2009 11;28(11):1375–82. 10.1007/s10096-009-0795-4 Epub 2009 Aug 25. 19701815 PMC2772956

[6] van Cleef BA, Verkade EJM, Wulf MW. Buiting AG, Voss A, Huijsdens XW, et al Prevalence of livestock-associated MRSA in communities with high pig-densities in the Netherlands. PLoS ONE, 2010; 5(2): e9385 20195538 10.1371/journal.pone.0009385 PMC2828479

[7] Costelloe C, Metcalfe C, Lovering A, Mant D, Hay AD. Effect of antibiotic prescribing in primary care on antimicrobial resistance in individual persons: systematic review and meta analysis. BMJ 2010:340:c2096 10.1136/bmj.c2096 20483949

[8] Tenover FC, Goering RV. Methicillin-resistant *Staphylococcus aureus* strain USA300: origin and epidemiology. J Antimicrob Chemother. 2009;64(3):441–6. 10.1093/jac/dkp241 19608582

[9] Wassenberg WM, Bonten MJM. Net Nederlandse MRSA-beleid kan en moet anders. Ned Tijdschrift Geneeskunde. 2010 13 11; 154 (45). 21118588

[10] Friedrich AW, Daniels-Haardt I, Köck R, Verhoeven F, Mellmann A, Harmsen D, et al EUREGIO MRSA-net Twente/Münsterland-a Dutch-German cross-border network for the prevention and control of infections caused by methicillin-resistant *Staphylococcus aureus* . Eurosurveillance 2008 8 28;13(35). pii: 18965 18761882 10.2807/ese.13.35.18965-en

[11] Malhotra-Kumar S, Lammens C, Coenen S, Van Herck K, Goossens H. Impact of azithromycin and clarithromycin therapy on pharyngeal carriage of macrolide-resistant streptococci among healthy volunteers: A randomized, double-blind, placebo-controlled study. Lancet 2007;369:482–90. 17292768 10.1016/S0140-6736(07)60235-9

[12] Mellmann A, Friedrich AW, Rosenkötter N, Röthganger J, Karch H, Reintjes R, et al Automated DNA sequence-based early warning system for the detection of methicillin-resistant *Staphylococcus aureus* outbreaks. Plosmed.2006 3;3(3):e33 10.1371/journal.pmed.0030033 PMC1325475 16396609

[13] Layer F, Cuny C, Strommenger B, Werner G, Witte W. Bundesgesundheitsblatt Gesundheitsforschung Gesundheitsschutz. 2012 11; 55(11–12):1377–86. 10.1007/s00103-012-1560-x In German. 23114436

[14] den Heijer CDJ, van Bijnen EDE, Paget WJ, Pringle M, Goossens H, Bruggeman CA, et al Prevalence and resistance of commensal *Staphylococcus aureus*, including meticillin-resistant *Staphylococcus aureus*: a European cross-sectional study. The Lancet Infectious Diseases, The Lancet Infectious Diseases, Early Online Publication, 6 3 2013; 10.1016/S1473-3099(13)70036-7 23473661

[15] Adriaenssens N, Coenen S, Versporten A, Muller A, Minalu G, Faes C, et al European Surveillance of Antimicrobial Consumption (ESAC): outpatient antibiotic use in Europe (1997–2009). J Antimicrob Chemother. 2011 12;66 Suppl 6:vi3–12. 10.1093/jac/dkr453 22096064

[16] Dalhoff A, Schubert S. Dichotomous selection of high-level oxacillin resistance in Staphylococcus aureus by fluoroquinolones. Int J Antimicrob Agents. 2010 9;36(3):216–21. 10.1016/j.ijantimicag.2010.04.014 Epub 2010 Jul 14. 20630710

[17] World Bank. Available: http://databank.worldbank.org/ddp/home.do. Accessed 2 April 2013.

[18] van Cleef BA, Kluytmans JA, van Benthem BH, Haenen A, Monen J, Daniels-Haardt I, et al Cross border comparison of MRSA bacteraemia between The Netherlands and North Rhine-Westphalia (Germany): a cross-sectional study. PLoS One. 2012;7(8):e42787 10.1371/journal.pone.0042787 Epub 2012 Aug 3. 22880109 PMC3411841

[19] European Centre for Disease Prevention and Control. Antimicrobial resistance interactive database: EARS-Net. Available: http://www.ecdc.europa.eu/en/healthtopics/antimicrobial_resistance/database/Pages/table_reports.aspx. Accessed 12 February 2014.

[20] Köck R, Brakensiek L, Mellmann A, Kipp F, Henderikx M, Harmsen D, et al Cross-border comparison of the admission prevalence and clonal structure of meticillin-resistant *Staphylococcus aureus* . J Hosp Infect. 2009 4;71(4):320–6. 10.1016/j.jhin.2008.12.001 Epub 2009 Feb 6. 19201056

[21] Bode LG, Wertheim HF, Kluytmans JA, Bogaers-Hofman D, Vandenbroucke-Grauls CM, Roosendaal R, et al Sustained low prevalence of meticillin-resistant Staphylococcus aureus upon admission to hospital in The Netherlands. J Hosp Infect. 2011 11;79(3):198–201. 10.1016/j.jhin.2011.05.009 Epub 2011 Jul 16. 21763031

[22] Wulf MW, Verduin CM, van Nes A, Huijsdens X, Voss A. Infection and colonization with methicillin resistant *Staphylococcus aureus* ST398 versus other MRSA in an area with a high density of pig farms. Eur J Clin Microbiol Infect Dis. 2012 1;31(1):61–5. 10.1007/s10096-011-1269-z Epub 2011 May 1. 21533878

[23] Wulf M, Voss A. MRSA in livestock animals-an epidemic waiting to happen? Clin Microbiol Infect. 2008 6;14(6):519–21. 10.1111/j.1469-0691.2008.01970.x Epub 2008 Mar 4. 18325034

[24] Monnet D. Methicillin-resistant Staphylococcus aureus and its relationship to antimicrobial use: possible implications for control. Infect Control and Hosp Epidemiol 1998; 19: 552–9. 9758054 10.1086/647872

[25] Tacconelli E, De AG, Catalldo MA, Pozzi E, Cauda R. Does antibiotic exposure increase the risk of methicillin-resistant *Staphylococcus aureus* (MRSA) isolation? A systematic reviw and meta-analysis. J Antimicrob Chemother 2008; 61: 26–38. 17986491 10.1093/jac/dkm416

[26] Statline Centraal Bureau voor de Statistiek. Beroepsbevolking naar bedrijf en persoonskenmerken (2008–2011). Available: http://statline.cbs.nl/StatWeb/publication/?VW=T&DM=SLNL&PA=80470NED&D1=0,2-4,13-18&D2=45&D3=0&D4=a&HD=121218-1539&HDR=T&STB=G2,G1,G3 Accessed 12 November 2012.

[27] Destatis Statistisches Bundesamt 2013.Gesundheitspersonal nach Berufen 2011. Available: http://destatis.de/DE/ZahlenFakten/GesellschaftStaat/Gesundheit/Gesundheitspersonal/Tabellen/Berufe.html. Accessed 12 November 2012.

