# Supplementary material for: MRSA Carriage in Community Outpatients: A Cross-Sectional Prevalence Study in a High-Density Livestock Farming Area along the Dutch-German Border
Source: PLoS One. 2015 Nov 30;10(11):e0139589. doi: 10.1371/journal.pone.0139589 (PMC4664395; doi:10.1371/journal.pone.0139589)
Supplement: S1 File — (DOC) [file pone.0139589.s001.doc]

**Annex: PreMA study questionnaire**

# PART 1 FILLED OUT BY GENERAL PRACTITIONER

Current date

Antibiotic usage in the previous 3 month:

| Antibiotic name | Dosage | Number of days | Health reason |
| --- | --- | --- | --- |
| 1. |  |  |  |
| 2. |  |  |  |
| 3. |  |  |  |
| 4. |  |  |  |
| 5. |  |  |  |

**PART 2 FILLED OUT BY PATIENT**

***General***

1. Gender male female

2. Year of birth

***Living and working* Yes No Unknown**

3. Are you living at or near to:

a. a pig farm?

If yes, I live: at farm ≤5km ≥5km

b. a cattle farm?

>

If yes, I live: at farm ≤5km ≥5 km

c. a poultry farm?

If yes, I live: at farm ≤5km ≥5km

**Yes No**

4. Do you have one of the following occupations?

a. Livestock farming (e.g. pig farming)

b. Health care (e.g. hospital or nursing home)

5. Do you handle (touch) the following animals during your work (professional or hobby):

a. live pigs?

b. live cows / veal calves?

c. live poultry?

6. Have you handled one of the following kinds of **raw** **meat** in the past month: **Yes No**

a. pork

b. beef / veal

c. poultry

If yes: at work? at home?

**Yes No Unknown**

**Health**

7. Have you had contact with an MRSA positive person in the last year?

8. Were you admitted to a hospital in the last 6 months?

If yes, which country .....................................................................................

**Thank you very much for completing the questionnaire**
